# They Have the Ability to Say Yes or No: Providers' Perspectives on Veterans' Service Refusal as a Cause of Readmissions

**DOI:** 10.1093/geroni/igab046.1399

**Published:** 2021-12-17

**Authors:** Nytasia Hicks, Polly Noel, Lauren Penney, Jacqueline Pugh

**Affiliations:** 1 GRECC - STVHCS Department of Veterans Affairs, San Antonio, Texas, United States; 2 South Texas Veterans Health Care system, South Texas Veterans Health System, Texas, United States; 3 South Texas Veterans Health Care System, San Antonio, Texas, United States; 4 South Texas Veterans Health Care system, San Antonio, Texas, United States

## Abstract

Service refusal, where patients actively refuse clinicians’ recommendations for additional services needed to achieve safe and full recovery after discharge, is a key but often overlooked cause of readmissions. There is a dearth of literature on the extent of service refusal and providers’ (e.g. clinicians, nurses, social workers) observations regarding how to deal with these situations. As part of a larger, 10 VA site organizational case study of readmission, semi-structured interviews exploring causes of readmission were conducted with 21-41 staff members at each site (n=314). 41 providers identified Veteran service refusal and decision-making as causes of readmission. Providers acknowledged the need to honor patient autonomy/self-determination in decisions while at the same time worrying about potential adverse outcomes. Incongruence between Veterans’ and providers’ perceptions (especially for capacity for self-care), goals, and discharge plans was also cited as a factor in service refusal. Frustration was also raised about initial acceptance of service followed by refusal at time of service delivery. Providers also felt readmissions increased even further when combined with lack of or inadequate caregiving arrangements/family support, lack of cognitive capacity, homelessness, or home care affordability. Findings point to the need for interventions to evaluate congruence between provider and patient assessment of self-care capabilities and provide more in-depth goal setting and motivational interviewing techniques to help patients reach more realistic post-discharge care goals.

